# Effect of the Nintendo Ring Fit Adventure Exergame on Running Completion Time and Psychological Factors Among University Students Engaging in Distance Learning During the COVID-19 Pandemic: Randomized Controlled Trial

**DOI:** 10.2196/35040

**Published:** 2022-03-22

**Authors:** Yi-Syuan Wu, Wei-Yun Wang, Ta-Chien Chan, Yu-Lung Chiu, Hung-Che Lin, Yu-Tien Chang, Hao-Yi Wu, Tzu-Chi Liu, Yu-Cheng Chuang, Jonan Wu, Wen-Yen Chang, Chien-An Sun, Meng-Chiung Lin, Vincent S Tseng, Je-Ming Hu, Yuan-Kuei Li, Po-Jen Hsiao, Chao-Wen Chen, Hao-Yun Kao, Chia-Cheng Lee, Chung-Bao Hsieh, Chih-Hung Wang, Chi-Ming Chu

**Affiliations:** 1 Graduate Institute of Life Sciences National Defense Medical Center Taipei Taiwan; 2 Department of Nursing Tri-Service General Hospital National Defense Medical Center Taipei Taiwan; 3 School of Nursing National Defense Medical Center Taipei Taiwan; 4 Research Center for Humanities and Social Sciences Academia Sinica Taipei Taiwan; 5 School of Public Health National Defense Medical Center Taipei Taiwan; 6 Department of Otolaryngology-Head and Neck Surgery Tri-Service General Hospital National Defense Medical Center Taipei Taiwan; 7 Graduate Institute of Medical Sciences National Defense Medical Center Taipei Taiwan; 8 School of Medicine National Defense Medical Center Taipei Taiwan; 9 School of Post-Baccalaureate Medicine Kaohsiung Medical University Kaohsiung Taiwan; 10 Department of Healthcare Administration and Medical Informatics Kaohsiung Medical University Kaohsiung Taiwan; 11 Department of Public Health College of Medicine Fu-Jen Catholic University New Taipei City Taiwan; 12 Department of Internal Medicine Tri-Service General Hospital National Defense Medical Center Taipei Taiwan; 13 Division of Gastroenterology and Hepatology Taichung Armed Forces General Hospital Taichung Taiwan; 14 Department of Biological Science and Technology National Yang Ming Chiao Tung University Hsinchu Taiwan; 15 Department of Computer Science National Yang Ming Chiao Tung University Hsinchu Taiwan; 16 Division of Colorectal Surgery, Department of Surgery Tri-Service General Hospital National Defense Medical Center Taipei Taiwan; 17 Division of Colorectal Surgery Department of Surgery Taoyuan Armed Forces General Hospital Taoyuan Taiwan; 18 Department of Biomedical Sciences and Engineering National Central University Taoyuan Taiwan; 19 Division of Nephrology Department of Internal Medicine Taoyuan Armed Forces General Hospital Taoyuan Taiwan; 20 Department of Life Sciences National Central University Taoyuan Taiwan; 21 Division of Nephrology, Department of Medicine Fu-Jen Catholic University Hospital School of Medicine, Fu-Jen Catholic University New Taipei City Taiwan; 22 Division of Nephrology, Department of Internal Medicine Tri-Service General Hospital National Defense Medical Center Taipei Taiwan; 23 Trauma and Critical Care Service, Department of Surgery Kaohsiung Medical University Hospital Kaohsiung Medical University Kaohsiung Taiwan; 24 Department of Medical Informatics Tri-Service General Hospital National Defense Medical Center Taipei Taiwan; 25 Division of General Surgery, Department of Surgery Tri-Service General Hospital National Defense Medical Center Taipei Taiwan; 26 Big Data Research Center College of Medicine Fu-Jen Catholic University New Taipei City Taiwan; 27 Department of Public Health Kaohsiung Medical University Kaohsiung Taiwan; 28 Department of Public Health China Medical University Taichung Taiwan

**Keywords:** exergaming, cardiac force index, running, physical activity, sleep quality, mood disorders, digital health, physical fitness, Nintendo Ring Fit Adventure, COVID-19 pandemic

## Abstract

**Background:**

The COVID-19 outbreak has not only changed the lifestyles of people globally but has also resulted in other challenges, such as the requirement of self-isolation and distance learning. Moreover, people are unable to venture out to exercise, leading to reduced movement, and therefore, the demand for exercise at home has increased.

**Objective:**

We intended to investigate the relationships between a Nintendo Ring Fit Adventure (RFA) intervention and improvements in running time, cardiac force index (CFI), sleep quality (Chinese version of the Pittsburgh Sleep Quality Index score), and mood disorders (5-item Brief Symptom Rating Scale score).

**Methods:**

This was a randomized prospective study and included 80 students who were required to complete a 1600-meter outdoor run before and after the intervention, the completion times of which were recorded in seconds. They were also required to fill out a lifestyle questionnaire. During the study, 40 participants (16 males and 24 females, with an average age of 23.75 years) were assigned to the RFA group and were required to exercise for 30 minutes 3 times per week (in the adventure mode) over 4 weeks. The exercise intensity was set according to the instructions given by the virtual coach during the first game. The remaining 40 participants (30 males and 10 females, with an average age of 22.65 years) were assigned to the control group and maintained their regular habits during the study period.

**Results:**

The study was completed by 80 participants aged 20 to 36 years (mean 23.20, SD 2.96 years). The results showed that the running time in the RFA group was significantly reduced. After 4 weeks of physical training, it took females in the RFA group 19.79 seconds (*P*=.03) and males 22.56 seconds (*P*=.03) less than the baseline to complete the 1600-meter run. In contrast, there were no significant differences in the performance of the control group in the run before and after the fourth week of intervention. In terms of mood disorders, the average score of the RFA group increased from 1.81 to 3.31 for males (difference=1.50, *P*=.04) and from 3.17 to 4.54 for females (difference=1.38, *P*=.06). In addition, no significant differences between the RFA and control groups were observed for the CFI peak acceleration (CFIPA)_walk, CFIPA_run, or sleep quality.

**Conclusions:**

RFA could either maintain or improve an individual’s physical fitness, thereby providing a good solution for people involved in distance learning or those who have not exercised for an extended period.

**Trial Registration:**

ClinicalTrials.gov NCT05227040; https://clinicaltrials.gov/ct2/show/NCT05227040

## Introduction

The global COVID-19 outbreak has changed people’s lifestyles [1], forcing them to self-isolate or engage in distance learning. These conditions have resulted in physical inactivity and sedentary behavior, thus introducing health risks [2,3]. Although research on the effects of COVID-19 on physical inactivity is still in the early stages [4], it is expected that people’s sedentary behavior will increase due to long hours of working from home. Some researchers have indicated that the metabolic equivalent of prolonged television or computer use is approximately 1.0 to 1.5 metabolic equivalent of task [5]. The Physical Activity Guidelines Advisory Committee Scientific Report published in 2018 revealed that sedentary behavior was directly related to mortality and morbidity associated with cardiovascular disease as well as the incidence of type 2 diabetes, colorectal cancer, and lung cancer [6,7]. Therefore, a physical training program that can be performed at home is necessary.

With technological development and innovation, exergaming has become a new option for fitness training, education, and health maintenance [8]. Exergaming is defined as a type of exercise that integrates different modes of digital games into physical activities. It offers a new way to perform physical activity at home without space restrictions being an issue. Moreover, exergaming has proved a successful business model [9]. Studies have shown that exergaming not only helps players perform light-to-moderate–intensity exercises [9,10] but also enhances players’ self-efficacy and willingness to engage in physical activities [11]. In 2007, Nintendo used Wii Fit (Nintendo Co, Ltd) in the rehabilitation of patients with Parkinson disease and stroke [12,13]. In addition, some research indicated that exergames demonstrated a positive effect on sleep quality shown by the Chinese version of the Pittsburgh Sleep Quality Index (CPSQI) score and mood disorders shown by the 5-item Brief Symptom Rating Scale (BSRS-5) score [14,15], whereas other studies found that exergaming could alleviate the symptoms of chronic low back pain [8]. Despite the existence of studies on the effect of exergaming on health maintenance or psychological improvement, it is still necessary to conduct extensive investigations on the changes or improvements of physical fitness among students after performing exergames.

In this study, the physical condition during exercise was monitored using a new indicator developed by Hsiao et al, namely a novel cardiac force index (CFI); CFI = weight × activity/heart rate per second (HR), which was calculated and monitored using a noninvasive device. The authors reported that this index was positively correlated with running performance [16]. Subsequently, another study suggested that the CFI was positively correlated with the G tolerance of pilots [17]. The purpose of this study was to observe changes in the CFI after performing exergames. In addition, this study modified the original CFI formula and converted the index into centimeters per beat (CMPB), which was presented as the distance of body movement during the running per heartbeat and was expressed in centimeters [18].

Therefore, assuming that the Ring Fit Adventure (RFA; Nintendo Co, Ltd) exergame is an effective and useful tool, this study aimed to explore the following: (1) whether RFA could improve physical fitness and reduce the completion time for a 1600-meter run; (2) whether RFA could improve sleep quality and mood disorders; and (3) whether the new CMPB index could provide results similar to those of the CFI and be suitable for estimating run time.

## Methods

### Study Design

This randomized prospective study lasted 4 weeks. The participants were voluntarily recruited and divided into an intervention and a control group. Taiwan’s Ministry of Education has formulated health-related physical fitness standards for adolescents aged 7 to 23 years [19]. Its safety and reliability have been verified [19], and therefore, we selected a 1600-meter running test. Before the start of the study, the participants were provided with a comprehensive explanation of the research content, and those who were willing to participate provided written informed consent. In addition, all the data were collected, analyzed, and stored anonymously. The study was approved by the Trial Committee of the Tri-Service General Hospital in Taiwan (approval number: C202005175; trial registration number: NCT05227040).

### Participants

The study period was from January to April 2021. The inclusion criteria were as follows: (1) students who were over 20 years old and did not have chronic diseases; (2) students who were able to complete the 1600-meter outdoor run before and after the intervention; (3) students who understood and agreed with the research purpose and signed the consent form; and (4) students who used wearable devices correctly.

### Sample and Randomization

**Figure 1:**
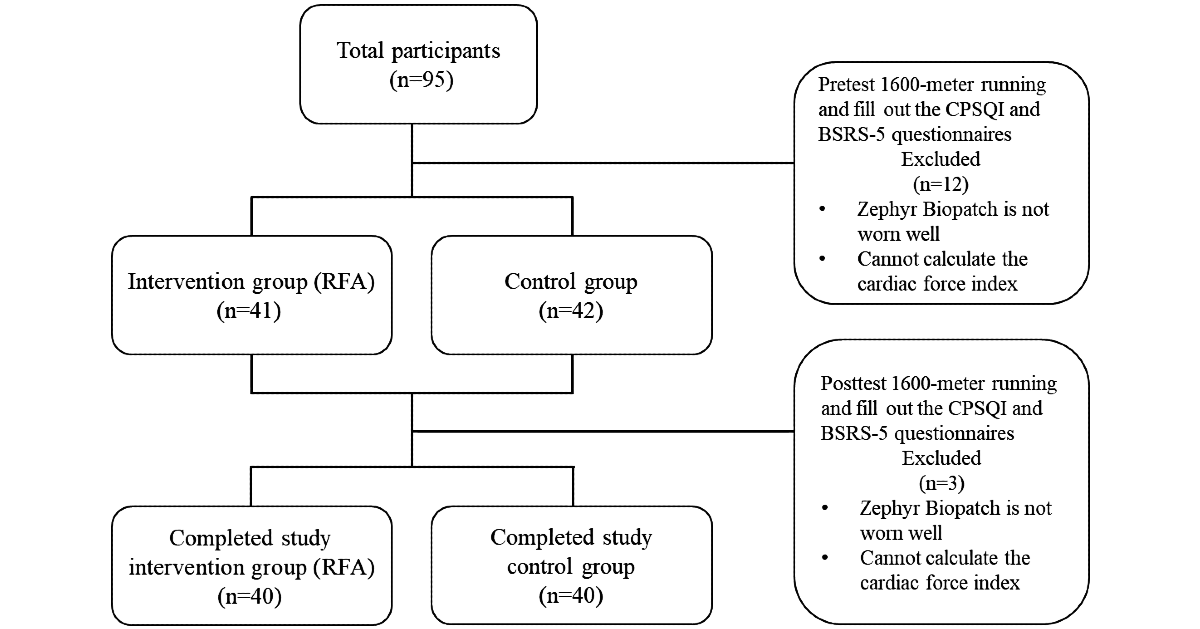
Flowchart showing enrollment of study participants. BSR-5: 5-item Brief Symptom Rating Scale; CPSQI: Chinese version of the Pittsburgh Sleep Quality Index; RFA: Ring Fit Adventure.

This randomized prospective study was conducted with 1 experimental group and 1 control group. A total of 95 participants were recruited for this study. However, 12 participants were excluded from the pretest because their CFI could not be calculated (Based on the Zephyr BioPatch Monitoring Device for Human Performance manual, the values of the HR confidence value lower than 20% indicated data were unreliable, and they were automatically deleted) [20]. Randomization was conducted using the simple random sampling method. After 4 weeks, during the posttest running test, the CFI of 3 participants could not be fully calculated, and hence, these participants were excluded. Finally, a total of 80 participants in the intervention group and control group completed the study (Figure 1).

### Intervention

**Figure 2:**
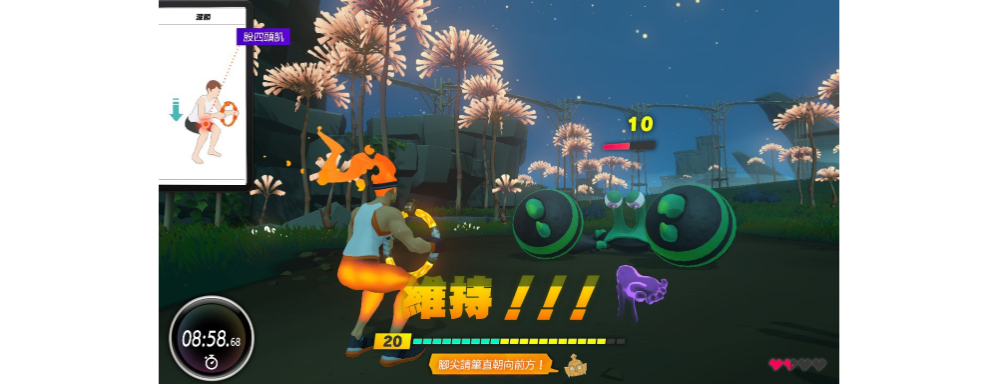
Adventure mode screen of Nintendo Ring Fit Adventure.

Participants in the RFA group were required to exercise for 30 minutes 3 times per week (in adventure mode) (Figure 2) for 4 weeks. The initial exercise intensity was set according to the instructions given by the virtual coach during the first game and was gradually adjusted according to the game instructions. The research team continued to track the RFA group subjects and encouraged the completion of 4 weeks of physical activity training.

In contrast, no intervention was introduced to the control group participants, who maintained their regular habits during the study period.

#### RFA of Nintendo Switch

RFA is a role-playing fitness exergame that uses a Ring-Con attached to a Joy-Con. The controller is equipped with high-precision sensors that can detect and digitize the player’s movements. Ring-Con is a resistance training device that allows users to jump over obstacles or attack objects by stretching or squeezing them. In addition, an infrared sports camera monitors the changes in the player’s heart rate. The game determines the optimal exercise intensity for the player and makes fine adjustments based on physiological feedback [8]. With an adjustable exercise intensity, RFA may be suitable for all age groups.

During battle scenes, players are required to defeat the enemies using a combination of different physical activities. Although the amount of exercise depends on the specific exercise intensity chosen by the player, it is generally set at 4 to 6 metabolic equivalents [21]; a previous study found that the exercise intensity of Wii Sports was between 3 and 5 metabolic equivalents [22].

### Measurements

#### CPSQI Score

The Pittsburgh Sleep Quality Index can be used to assess an individual’s sleep quality at a 1-month interval and extract quantitative and qualitative information about sleep based on sleep quality data [23]. The CPSQI developed by Tsai et al in 2005 showed superior validity, consistency (Cronbach α=.75-.85) [24,25], and test-retest reliability (0.85 for the 14- and 21-day intervals).

The scale consists of 7 factors (subjective sleep quality, sleep latency, sleep duration, habitual sleep efficiency, sleep disturbances, use of sleep-promoting medication, and daytime dysfunction). The total score of the scale ranges between 0 and 21 and is obtained by summing the individual scores. A higher score indicates poorer sleep quality. Generally, people with a CPSQI score greater than 5 are considered to have poor sleep quality [24].

#### 5-item BSRS-5 Score

Studies have indicated that the BSRS-5 is an effective tool that can screen the onset of mental illness and suicidal ideation [26,27]. The BSRS-5 can also be used to investigate the mood disorder of an individual within a week [28]. The scale includes five items: (1) having trouble falling asleep; (2) feeling tense or high-strung; (3) feeling easily annoyed or irritated; (4) feeling depressed or being in a low mood; (5) feeling inferior to others; and an additional question on suicidal ideation. Participants are required to answer these questions on a 5-point Likert scale (0-4) [26] and then the total score is calculated as the sum of the individual scores. A score >6 indicates the onset of mental illness or mood disorder. In addition, participants who have a score >2 on suicidal ideation are recommended to seek professional consultation or mental health care [29].

#### CFI Measurement

The CFI is used to measure the heart condition during exercise; it is patented in the United States (US 9566010 B2) [30] and Taiwan (I546051) [31] and can be used to calculate the amount of work done by the heart for body movement per heartbeat per second. When combining this index with acceleration, the individual status can be analyzed during physical activity; this index is a feedback indicator for monitoring exercise intensity and load [16]. CFI=weight × activity/heart rate [16]. The CFI can also be converted to CMPB (cm/beat), namely the distance (in cm) the body can move during each heartbeat. The index can be used to indicate changes in distance during each heartbeat, and centimeter is a common unit, which is very convenient for people to understand [18].

#### Wearable Device

**Figure 3:**
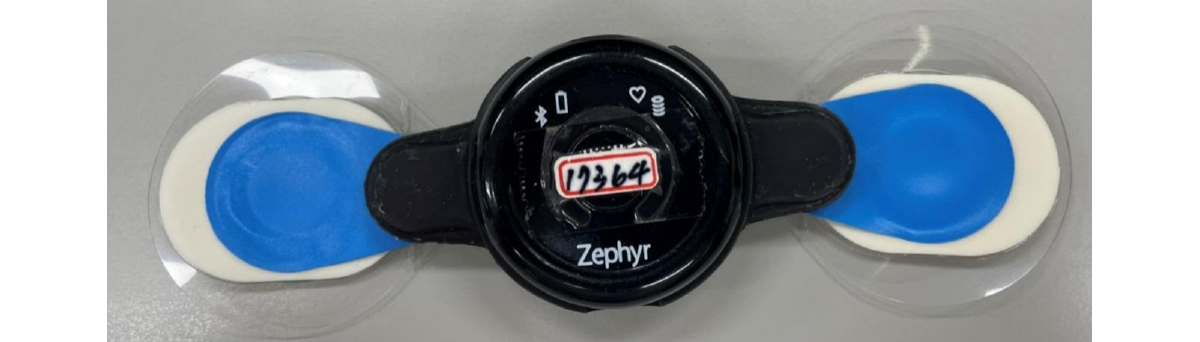
Zephyr BioPatch wearable device.

We used a Zephyr BioPatch loaded with the Zephyr BioHarness 3.0 module (Zephyr Technology Corporation). In addition, the Ambu BlueSensor T, an electrocardiogram (ECG) patch (Ambu A/S, Ballerup) [32], was attached to the participants’ apical area. In addition, when attached to the BioPatch (Figure 3), it could measure the potential changes in the skin through the bilateral ECG patches and consequently record the heart rate and respiratory rate.

The Zephyr BioPatch is a device approved by the US Food and Drug Administration for medical and clinical applications [20,33]. It can provide reliable heart rate measurements [34]. Zephyr BioHarness 3.0 is noninvasive and consists of a 3-axis gyroscope and an accelerometer to distinguish between the x-, y-, and z-axes. Moreover, it can measure and estimate basic physiological parameters, such as the heart rate, respiratory rate, acceleration, maximum oxygen consumption, and temperature. The data can be displayed in real time through Bluetooth and are stored in the memory of the device, allowing access for statistical analysis [20,33].

### Data Collection

Before the pretest and posttest, the participants’ basic demographic information, including age, sex, height, weight, neck circumference, waist circumference, smoking status (yes or no), alcohol consumption status (yes: 2 to 3 times a week or at least once a week; or no), milk intake (yes or no), whether the participants consumed 3 servings of vegetables and 2 servings of fruit per week, exercise habits (4 to 6 times a week, 1 to 3 times a week, or none), sleep quality (CPSQI), and mood disorder (BSRS-5 score), was collected.

Prior to the 1600-meter outdoor run, participants were required to wear the Zephyr BioPatch loaded with the Zephyr BioHarness 3.0 module. In addition, the Ambu BlueSensor T [32] was attached to the participants’ apical area (Figure 3).

After the participants completed the 1600-meter run, the BioHarness 3.0 module was removed from the BioPatch, and the physiological data were extracted. The data, which were binned per second, were then calculated and saved in Excel sheets (Microsoft Corporation). Due to the large amount of data, the method described by Hsaio et al was adopted for data processing [16]. Subsequently, a member of the research team further reviewed the physiological data collected by the BioHarness 3.0 module and calculated the CFI and CMPB values. CFI is an established method for monitoring heart function and workload during physical activity [16]. Its advantages include being noninvasive, wireless, and the ability for real-time monitoring through mobile devices [16].

The definitions of the BioHarness 3.0 parameters are listed below [16,18]:

1. CFI peak acceleration (CFI_PA): It was the peak acceleration per second divided by the number of heartbeats per second, both of which were estimated and measured by BioHarness 3.0.

2. Heart rate: It is defined as the number of heartbeats per second measured by the sensors during exercise. With BioHarness 3.0, the heart rate data were recorded in beats per minute (bpm), and the result was the average value of the data over 1 second.

3. CFI_PA while walking (CFI_PA_walk): This was the peak acceleration per second divided by the number of heartbeats per second while the participant was walking.

4. CMPB while walking (CMPB_walk): It was the movement in centimeters per second divided by the number of heartbeats per second while the participant was walking.

5. CFI_PA while running (CFI_PA_run): This was the peak acceleration per second divided by the number of heartbeats per second while the participant was running.

6. CMPB while running (CMPB_run): This was the average value of the CMPB_run calculated using the start of the run to the end of the run (movement in centimeters per second divided by the number of heartbeats per second while the participant is running).

### Statistical Analysis

The Windows version of the SPSS 28.0 software (IBM Corp) was used for the statistical analysis. According to descriptive statistics, categorical data are expressed in numbers and percentages, whereas continuous variables are expressed as averages and SDs. We conducted independent sample *t* tests and chi-square tests to confirm group differences.

Subsequently, the paired *t* test was conducted to analyze the differences between the completion time for the 1600-meter run in seconds, CFI, CMPB, CPSQI, and mood disorders between the pretest and posttest. We determined that the data may not be normally distributed, and therefore, we additionally used the Wilcoxon signed rank test for analysis.

Alternatively, generalized estimating equations (GEEs) were adopted to predict important factors influencing the running time. The significance level was set at 2-tailed *P*<.05.

## Results

### Participant Demographics

The study was completed by 80 participants aged 20 to 36 years (mean 23.2, SD 2.96 years), and both groups were composed of 40 individuals. The RFA group comprised 16 males and 24 females, with an average age of 23.75 (SD 3.58) years; the control group included 30 males and 10 females, with an average age of 22.65 (SD 2.08) years. None of the participants were smokers; 51 participants (64%) exercised 1 to 3 times a week.

In addition, there were no significant differences in the participants’ age, BMI, neck circumference, waist circumference, smoking status, and alcohol consumption status; there were also no differences regarding whether they drank milk, consumed 3 servings of vegetables and 2 servings of fruit per week, and the exercise habits between the RFA and the control groups. The detailed demographics of the participants are listed in Table 1.

**Table 1:** Demographics of the participants in each group (N=80)^a^.

Variable			Total		RFA^b^ group		Control group		*χ*^2^/t (*df*)			*P* value	
Age (years), mean (SD)			23.20 (2.96)		23.75 (3.58)		22.65 (2.08)		–1.68 (78)			.1	
BMI (kg/m^2^), mean (SD)			21.96 (2.69)		21.80 (2.77)		22.13 (2.63)		0.54 (78)			.59	
Neck circumference, mean (cm) (SD)			33.96 (2.76)		33.40 (2.68)		34.50 (2.75)		1.80 (78)			.08	
Waist (cm) mean (SD)			74.53 (7.95)		73.67 (7.77)		75.38 (8.12)		0.96 (78)			.34	
**Gender, n (%)**										10.02 (1)			.002
	Female	34		24 (70.6)		10 (29.4)							
	Male	46		16 (34.8)		30 (65.2)							
**Smoking status, n (%)**										N/A^c^			N/A
	Yes	0		0 (0)		0 (0)							
	No	80		40 (50)		40 (50)							
**Alcohol, n (%)**										0.35 (2)			.84
	2-3 times a week	3		1 (33.3)		2 (66.7)							
	At least once a week	6		3 (50)		3 (50)							
	None	71		36 (50.7)		35 (49.3)							
**Drinking milk, n (%)**										3.41 (1)			.07
	Yes	30		21 (42)		29 (58)							
	No	50		19 (63.3)		11 (36.7)							
**Eating at least 3 servings of vegetables and 2 of fruits, n (%)**										0.56 (1)			.46
	Yes	72		35 (48.6)		37 (51.4)							
	No	8		5 (62.5)		3 (37.5)							
**Exercise habits, n (%)**										0.91 (2)			.64
	4-6 times a week	15		6 (40)		9 (60)							
	1-3 times a week	51		26 (51)		25 (49)							
	None	14		8 (57.1)		6 (42.9)							

^a^The age, BMI, neck circumference, and waist circumference were used in the *t* test and the rest of the variables were used in the chi-square tests.

^b^RFA: Ring Fit Adventure.

^c^N/A: not applicable.

### Intervention Outcomes

#### Comparison of Running Time

After 4 weeks of the intervention, the time taken by the RFA group to complete the run (in seconds) decreased significantly from 611.75 seconds to 591.96 seconds for females (difference=–19.79; *P*=.03) and from 489.5 seconds to 466.94 seconds for males (difference=–22.56; *P*=.03) (Table 2).

In contrast, the time taken by the control group to complete the run (in seconds) increased from 483.6 seconds to 491.83 seconds for males and decreased from 598.3 seconds to 583.8 seconds for females. However, the differences between the preintervention and postintervention periods were not significant.

#### Heart Performance Indices

After 4 weeks of the intervention, CFIPA_run increased from 13.95 to 15.02 in females (difference=1.07, *P*=.11) and 16.54 to 17.38 in males (difference=0.845, *P*=.22). CMPB_run increased from 83.68 to 90.09 in females (difference=6.41, *P*=.11) and increased from 99.21 to 104.28 in males (difference=5.07, *P*=.22) (Table 2). In addition, no significant differences between the RFA and control groups were observed for CFIPA_ walk, CMPB_ walk, CFIPA_ run, or CMPB_ run.

#### Psychological Factors

In terms of mood disorders, the average score of the RFA group increased from 1.81 to 3.31 for males (difference=1.50, *P*=.04) and from 3.17 to 4.54 for females (difference=1.38, *P*=.06) (Table 2). The sleep quality score (CPSQI) increased from 4.81 to 5.56 (difference=0.75, *P*=.19) for males and from 5.75 to 6.21 (difference=0.46, *P*=.38) for females, but there was no statistically significant difference. In addition, no significant differences between the control groups were observed for sleep quality (CPSQI) or mood disorders.

**Table 2:** Comparison of run time, cardiac force index, and psychological factors after 4 weeks of intervention.

Group				Pretest		Posttest		Difference		*P* value^a^
**RFA^b^ group**										
	**Females**									
		Running seconds, mean (SD)	611.75 (92.92)		591.96 (87.39)		–19.79 (37.93)		.03	
		CFIPA^c^_ walk, mean (SD)	6.28 (1.25)		6.22 (1.75)		–0.06 (1.72)		.55	
		CMPB^d^_ walk, mean (SD)	37.66 (7.49)		37.29 (10.51)		–0.67 (10.35)		.55	
		CFIPA_ run, mean (SD)	13.95 (3.59)		15.02 (4.77)		1.07 (3.24)		.11	
		CMPB_ run, mean (SD)	83.68 (21.56)		90.09 (28.64)		6.41 (19.45)		.11	
		Sleep quality, mean (SD)	5.75 (3.40)		6.21 (2.64)		0.46 (2.23)		.38	
		Mood disorder, mean (SD)	3.17 (2.71)		4.54 (4.08)		1.38 (3.06)		.06	
**Males**										
		Running seconds, mean (SD)	489.50 (65.53)		466.94 (54.17)		–22.56 (36.11)		.03	
		CFIPA_ walk, mean (SD)	6.37 (0.81)		6.07 (1.07)		–0.30 (1.05)		.28	
		CMPB_ walk, mean (SD)	38.24 (4.85)		36.44 (6.43)		–1.80 (6.33)		.28	
		CFIPA_ run, mean (SD)	16.54 (2.84)		17.38 (2.82)		0.845 (1.95)		.22	
		CMPB_ run, mean (SD)	99.21 (17.07)		104.28 (16.93)		5.07 (11.69)		.22	
		Sleep quality, mean (SD)	4.81 (2.56)		5.56 (2.71)		0.75 (1.95)		.19	
		Mood disorder, mean (SD)	1.81 (2.90)		3.31 (3.98)		1.50 (2.71)		.04	
**Control group**										
	**Females**									
		Running seconds, mean (SD)	598.30 (79.38)		583.80 (92.06)		–14.50 (39.96)		.24	
		CFIPA_ walk, mean (SD)	6.50 (1.03)		6.98 (2.93)		0.48 (2.76)		.65	
		CMPB_ walk, mean (SD)	39.01 (6.16)		41.87 (17.58)		2.86 (16.59)		.65	
		CFIPA_ run, mean (SD)	13.32 (3.24)		14.12 (4.66)		0.81 (2.37)		.14	
		CMPB_ run, mean (SD)	79.90 (19.46)		84.74 (27.94)		4.85 (14.21)		.14	
		Sleep quality, mean (SD)	3.90 (3.57)		3.80 (2.10)		0.70 (2.32)		.34	
		Mood disorder, mean (SD)	5.80 (2.57)		6.50 (2.91)		–0.10 (3.54)		.86	
	**Males**									
		Running seconds, mean (SD)	483.60 (47.92)		491.83 (51.97)		8.23 (40.05)		.33	
		CFIPA_ walk, mean (SD)	7.17 (1.20)		7.15 (1.85)		–0.02 (1.50)		.45	
		CMPB_ walk, mean (SD)	43.03 (7.23)		42.90 (11.10)		–0.12 (9.00)		.45	
		CFIPA_ run, mean (SD)	18.48 (4.37)		18.81 (3.45)		0.33 (2.43)		.21	
		CMPB_ run, mean (SD)	110.89 (26.22)		112.85 (20.70)		1.95 (14.59)		.21	
		Sleep quality, mean (SD)	5.93 (2.74)		5.37 (2.77)		–0.57 (2.50)		.26	
		Mood disorder, mean (SD)	2.90 (3.04)		4.07 (2.91)		1.17 (4.09)		.15	

^a^ obtained from the Wilcoxon signed rank test.

^b^CFIPA: cardiac force index peak acceleration.

^c^CMPB: centimeters per beat.

^d^RFA: Ring Fit Adventure.

### Predicted Physical Factors Affecting the Completion of the 1600-Meter Run

We used linear regression to select the variables with a significance level at *P*<.05. A prediction model was established using GEEs, and the BioHarness 3.0 parameters were introduced to identify factors that affected the time taken by the 80 participants to complete the run. The explanatory power of the model was 66.3% (*R^2^*=0.663). The results indicated that sex, exercise habits, alcohol consumption status, CMPB_ run, age, and BMI were important factors that affected the time taken for completing the run. If variables such as the group, sex, age, sleep quality, mood disorder, BMI, neck circumference, and waist circumference were controlled, those who exercised 4 to 6 times per week took 48.02 seconds less to complete the run (95% CI –85.65 to –10.38), and compared to nondrinkers, those who consumed alcohol 4 to 6 times per week took 45.83 seconds more to complete the run (95% CI 6.64 to 85.01). Furthermore, each additional unit of CMPB_ run corresponded to a decrease of 0.89 seconds in run time (95% CI –1.37 to –0.4) (Table 3).

**Table 3:** Predicted physical factors affecting the completion of the 1600-meter run.

Variable				Beta (95% CI)		*P* value
**Group**						
	RFA^a^		–6.24 (–32.04 to 19.57)		.64	
	Control		N/A^b^		N/A	
**Gender**						
	Male		–96.10 (–125.25 to –66.94)		<.001	
	Female		N/A		N/A	
**Exercise habits**						
	4-6 times a week		–48.02 (–85.65 to –10.38)		.01	
	1-3 times a week		–25.88 (–57.20 to 5.45)		.11	
	None		N/A		N/A	
**Alcohol consumption**						
		2-3 times a week	45.83 (6.64 to 85.01)		.02	
		At least once a week	–1.66 (–42.28 to 38.96)		.94	
		None	N/A		N/A	
Age				3.68 (0.02 to 7.34)		.05
Sleep quality				2.13 (–0.86 to 5.13)		.16
Mood disorder				–1.58 (–4.20 to 1.03)		.24
CMPB^c^_ run				–0.89 (–1.37 to –0.40)		<.001
BMI				10.02 (3.17 to 16.87)		.004
Neck circumference				1.54 (–1.89 to 4.98)		.38
Waist				–0.23 (–1.37 to 0.91)		.69

^a^RFA: Ring Fit Adventure.

^b^N/A: not applicable.

^c^CMPB: centimeters per beat.

## Discussion

### Principal Findings

To our knowledge, this is the first study assessing the effect of a Nintendo RFA exergame on running completion time of adults after an intervention. As a study that incorporated a popular exergame (RFA) into the intervention implemented among students, in addition to assessing physical fitness and changes in the time it took to complete a run, this study also investigated the changes in the participants’ sleep quality and psychological factors related to mood disorder.

In the RFA group, after 4 weeks of intervention, the completion time for the 1600-meter run decreased significantly for male and female participants, which was consistent with the findings of a previous study [11]. For example, a meta-analysis of studies conducted with children and adolescents found that previous exergames, such as Wii Fit and Dance Revolution, demonstrated similar physiological benefits [11].

Another 3-group randomized controlled trial of obese teenagers aged between 15 and 19 years reported that those who were encouraged to cooperatively play Wii Fit for 40 to 60 minutes per day lost substantially more weight and had an elevated self-identity [35,36]. In addition, similar exergames on Xbox Kinect (Microsoft Corp) exhibited a positive effect on physical fitness and emotional cognition [37] and were proven to enhance the dynamic balance of the lower limbs [38]. Both these benefits were attributed to the fact that Xbox Kinect required users to actively move in a given space by making the tasks dynamic [39] and introduced training programs that promoted visual feedback and dynamic balance [40].

Furthermore, a study provided preliminary evidence that Wii Fit could encourage people to exercise, thereby alleviating fatigue and reducing their body weight, waist circumference, anxiety level, and overall pain intensity [41]. Among traditional training programs, high-intensity interval training and similar methods use short-term high-intensity exercises to increase cardiorespiratory fitness and maximum oxygen consumption [42]. Moreover, a relatively short exercise time can effectively improve aerobic metabolism [43,44].

However, it remains unknown whether individuals can sustain such exercises over an extended period. In contrast, by combining exercise with digital games, emerging exergames can integrate physical activities with cognitive tasks and facilitate players to engage in physical activity according to visual, audio, and sensational feedback [45]. In addition, its user stickiness is strong owing to the introduction of multiple levels. Although this study did not include elderly individuals as part of the study population, similar research indicated that their experience with exergames was positive [46]. Exergames could also be used for rehabilitation purposes, as a study suggested that rehabilitation through Wii Fit achieved satisfactory therapeutic effects [47]. A recent study that adopted RFA as an intervention found that after 8 weeks, the chronic low back pain of participants was effectively alleviated [8]. The high level of movement they provide and the reduced cost of interactive modern technology have made exergames increasingly popular [48]; therefore, their application in health promotion is considered a future trend [49].

### Influence of Exergames on Psychological Factors

The existing studies on the effects of exergames on social and psychological factors, such as sleep quality and mood disorders, are somewhat limited. Our research indicated no significant changes in sleep quality after the intervention. The CPSQI adopted in this study is a psychometrically reliable measure of sleep quality and sleep disorders in patients with primary insomnia [25]. Although a study found that 6 weeks of Xbox Kinect training could improve the sleep quality of elderly individuals and alleviate their anxiety symptoms [50], this study did not reach a similar conclusion.

Alternatively, the BSRS-5 scale used in this study to measure mood disorders can be used to assess psychological symptoms, including anxiety, depression, hostility, interpersonal sensitivity, and difficulty falling asleep [26,28,51]. The modified BSRS-5 was found to rapidly detect mood disorders in a previous Taiwanese study and showed high validity and reliability among the general public as well as patients with mental or medical diseases [26]. In this study, although the BSRS-5 scores of males (1.81 to 3.31) and females (3.17 to 4.54) in the RFA group increased considerably after the intervention, they did not reach the category of mild mood disorder (BSRS-5 score>6). At present, no studies have used the BSRS-5 to explore the effectiveness of exergames. Although a study suggested that exergames could alleviate anxiety symptoms [41], a different questionnaire was used.

Therefore, more relevant research may be required to provide further evidence. The increased mood disorders in this study were likely because the pretest was performed immediately before the Lunar New Year Festival, whereas the posttest was performed after the holiday and before the midterm examinations, during which the participants were possibly more stressed. Some researchers also pointed out that during the COVID-19 pandemic, even low-threshold and mild psychological reactions could provoke intense mood disorder reactions [52].

### Predicted Physical Factors Affecting the Completion of the 1600-Meter Run

This study proposed a new index, CMPB, based on the original CFI. CMPB refers to the distance (in cm) the body can move during each heartbeat, which makes it easier for the public to understand. Our study showed that sex, exercise habits, alcohol consumption status, CMPB_run, age, and BMI were important factors that affected an individual’s performance in the 1600-meter run. A previous study found that the older the person, the longer the run completion time [16], which was consistent with our findings. In addition, our study included exercise habits as a variable and found that compared to those who did not have an exercise habit, participants who regularly exercised 4 to 6 times a week required substantially less time to complete the 1600-meter run, which was consistent with the result of another study [53].

Moreover, we found that compared to nondrinkers, participants who consumed alcohol 2 to 3 times a week took significantly longer to complete the run, which was consistent with the findings of a previous study that focused on the correlation between alcohol consumption and exercise [54,55]. Furthermore, our study showed that each additional unit increase in CMPB_run reduced the time required to complete the run, and the difference was statistically significant. This was in line with the results of a CFI study [16], which indicated that CMPB could provide good predictions similar to those of CFI. However, further research is required to support this conclusion.

Finally, our study used the novel Zephyr BioPatch device. As a result, the participants were no longer required to wear a strap on the lower edge of the chest for the Zephyr BioHarness 3.0 module to take measurements, which previously prevented women from participating [16]. Therefore, the CMPB indicator allowed measurements to be taken among female participants and thus identify sex-specific differences.

### Strengths and Limitations

This study had several strengths. First, the study found that RFA provided an opportunity for indoor exercise during the COVID-19 pandemic [56]. In addition, users’ constant engagement in training and activities could be encouraged by different elements in the game. Second, aside from running performance, this study also investigated the effect of RFA on sleep quality and mood disorders. Third, a new index, CMPB, was proposed based on the original CFI. Hence, the CMPB allows for easier comprehension and shares the same important factors in predicting the time required to complete the run.

However, this study had some limitations. First, the participants were limited to military students (college, master, and doctoral students) in northern Taiwan. Second, the study did not explore potential interfering elements, such as lifestyle habits and environmental factors. In addition, healthy participants may have contributed to a plateau effect, as their performance was already close to an optimal level [14].

### Conclusions

This study found that training using RFA could maintain or improve users’ physical fitness. Therefore, RFA provides a good solution for people who engage in distance learning for a prolonged period or those who do not have sufficient time for exercise. Exergames not only serve as an alternative for exercising at home but also show the potential to evolve with societal changes.

## Appendix

Multimedia Appendix 1 CONSORT-EHEALTH checklist (V 1.6.1).

## Abbreviations

BSRS-5: 5-item Brief Symptom Rating Scale
CFI: cardiac force index
CFI_PA_walk: cardiac force index of peak acceleration while walking
CMPB_walk: centimeters per beat while walking
CFI_PA_run: cardiac force index of peak acceleration while running
CMPB_run: centimeters per beat while running
CPSQI: Chinese version of the Pittsburgh Sleep Quality Index
GEE: generalized estimating equation
RFA: Ring Fit Adventure
